# Semaglutide-associated drug-induced liver injury: a case report and review of the literature

**DOI:** 10.1093/omcr/omaf177

**Published:** 2025-09-28

**Authors:** Ian Kempster, Darren Fernandes, Mohammed S Saeed, Caroline Sharratt, Sara Benfield

**Affiliations:** The Department of Gastroenterology, Sherwood Forest Hospitals NHS Trust, Mansfield Road, Sutton-in-Ashfield, NG17 4JL, United Kingdom; The Department of Gastroenterology, Sherwood Forest Hospitals NHS Trust, Mansfield Road, Sutton-in-Ashfield, NG17 4JL, United Kingdom; The Department of Histopathology, Sherwood Forest Hospitals NHS Trust, Mansfield Road, Sutton-in-Ashfield, NG17 4JL, United Kingdom; The Department of Gastroenterology, Sherwood Forest Hospitals NHS Trust, Mansfield Road, Sutton-in-Ashfield, NG17 4JL, United Kingdom; The Department of Gastroenterology, Sherwood Forest Hospitals NHS Trust, Mansfield Road, Sutton-in-Ashfield, NG17 4JL, United Kingdom

**Keywords:** drug induced liver injury, acute liver failure, GLP-1 receptor agonists, Semaglutide

## Abstract

Semaglutide is a glucagon-like peptide-1 receptor agonist (GLP-1RA) used to manage type 2 diabetes and, since 2021, for weight loss in individuals with obesity or weight-related comorbidities. It works by enhancing insulin secretion, delaying gastric emptying and reducing appetite. Common side effects include hypoglycaemia, gastrointestinal disturbances, nausea, weight loss and cholelithiasis. While some studies have noted an association with acute kidney injury, reports of liver injury are rare. We present a rare case of drug-induced liver injury in a middle-aged female, associated with transient liver failure after semaglutide use. She presented one month after starting the medication with rapidly worsening liver function tests. Investigations, including a non-invasive liver screen, viral studies, ultrasound and CT imaging, revealed no clear cause. A liver biopsy supported the diagnosis of drug-induced liver injury. The patient improved with supportive treatment and withdrawal of semaglutide. This case underscores the importance of clinician awareness given its increasing, and often unregulated, use for weight loss.

## Introduction

Semaglutide is a diabetic medication which binds to glucagon-like peptide-1 receptors. It has been licensed for use in type 2 diabetes since 2017 [1]. GLP-1 receptor agonists (GLP-1RAs) work by delaying gastric emptying and reducing appetite. It is for this reason that they have recently been licensed in 2021 for weight loss in patients, alongside dietary measures and increased physical activity, for those with a BMI > 30 kg/m^2^ or > 27 kg/m^2^ with at least one weight-related comorbidity [1, 2].

Semaglutide is metabolised in plasma through proteolytic cleavage of the peptide backbone and beta-oxidation of the fatty acid side chain [3]. This process occurs throughout the body with no single organ being the primary site of elimination. As a result, Semaglutide is considered to have a good safety profile. Common side effects include hypoglycaemia, gastrointestinal (GI) disturbance, nausea, weight loss and cholelithiasis [1]. Its use has been associated with acute kidney injury [4], however hepatotoxicity is not listed as a side effect.

There are few reports of drug induced liver injury from GLP-1RAs in current literature. A 2022 cohort study comparing dipeptidyl peptidase-4 (DPP-4) with GLP-1 RAs of 106 310 patients found an increased risk of acute liver injury in female patients taking GLP-1RAs compared to patients taking DPP-4 inhibitors [5]. Currently there have not been any published cases of hepatic injury that has been recorded on the LiverTox Database. We therefore report an extremely rare and potentially fatal episode of drug induced liver injury related to Semaglutide use.

## Case description

A middle aged female patient was admitted complaining of severe abdominal pain and nausea. Her BMI was 30.1 kg/m^2^ and she reported mobility issues secondary to her weight. She was therefore started on semaglutide by an online pharmacy losing 10.2 kg in the month prior to presentation. Her past medical history included hypothyroidism, back pain, vitamin B12 deficiency and iron deficiency. She took levothyroxine 25 micrograms once daily and pregabalin 50 mg once daily for back pain but denied using any over the counter or herbal medications. Socially, she was a non-smoker, drank up to 4 units of alcohol per week and denied any illicit drug use.

On admission, she had a normal liver function test (LFTs), normal renal function and normal clotting. However, she was found to be hypoglycaemic with a blood glucose of 2.70 and her VBG showed a metabolic acidosis with normal lactate (Table 1). She was therefore admitted to hospital and initially treated with intravenous (IV) fluids, including an IV Glucose infusion and anti-emetics.

**Table 1:** Admission blood results.

Blood Test	Result
ALT	16
ALP	82
Bilirubin	7
INR	0.9
Creatinine	77
pH	7.230
Lactate	1.0
Blood Glucose	2.70

48 h later the patient developed worsening abdominal pain and derangement in her intrinsic and extrinsic liver function. Her ALT rose sharply from 16 to 546 and Bilirubin from 7 to 37. She developed encephalopathy, severe coagulopathy and was admitted to the Intensive Care Unit as her liver function tests continued to deteriorate and she was diagnosed with a life-threatening acute liver failure (Table 2).

**Table 2:** Trend in blood results following admission.

		First 5 days of admission				
Blood Test	Day 1	Day 2	Day 3	Day 4	Day 5	Day 26
ALT (U/l)	16	Not done	546	5989	9934	66
ALP (U/l)	82	Not Done	90	101	140	198
Bilirubin (umol/l)	7	Not done	37	69	79	128
INR	0.9	Not done	1.4	4.1	6.3	1.0
Creatinine (umol/l)	77	65	57	66	124	70
pH	7.230	7.350	7.440	-	7.170	7.420
Lactate (mmol/l)	1.0	0.7	2.7	-	17.1	2.0

She underwent a non-invasive liver screen which showed no obvious cause for her acute liver failure (Table 3).

**Table 3:** Non-invasive liver screen and imaging.

Test	Result
Alpha 1 Antitrypsin (g/l)	1.05 (0.9–2.0)
AAT Genotype	MM Genotype
Caeruloplasmin (g/l)	0.192 (0.15–0.45)
IgG (g/l)	8.96 (7–16)
IgA (g/l)	2.23 (0.7–4)
ANA	Negative
Gastric Parietal Cell Antibodies	Negative
AMA	Negative
Smooth muscle antibodies	Negative
Liver kidney microsomal antibodies	Negative
Hep B core total antibody	Not detected
Hep B surface antigen	Not detected
Hep C antibody	Not detected
Hep A IgM antibody	Not detected
Anti-Hep E Virus IgG and IgM	Not detected
Immunoglobulins	Negative
EBV IgM	Negative
EBV VCA IgG	Positive (evidence of past infection)
EBV DNA	Not detected
CMV IgG and IgM	Not detected
CMV DNA	Not detected
Toxoplasmosis	Negative
Parvovirus	Negative
Borrelia	Negative
Paracetamol Level	Normal
CT Angiogram Aorta	No dissection or aneuryms, no abnormality detected
Ultrasound Abdomen	Normal Appearances of liver, no vascular or biliary obstruction
Lumbar Puncture	Negative
CT Head	No Acute abnormality

During her stay on intensive care, she was managed supportively and N-acetyl cysteine was administered intravenously. She was also discussed with the local transplant centre in case of non-response. Fortunately, she began to recover and her liver function started to improve.

**Figure 1:**
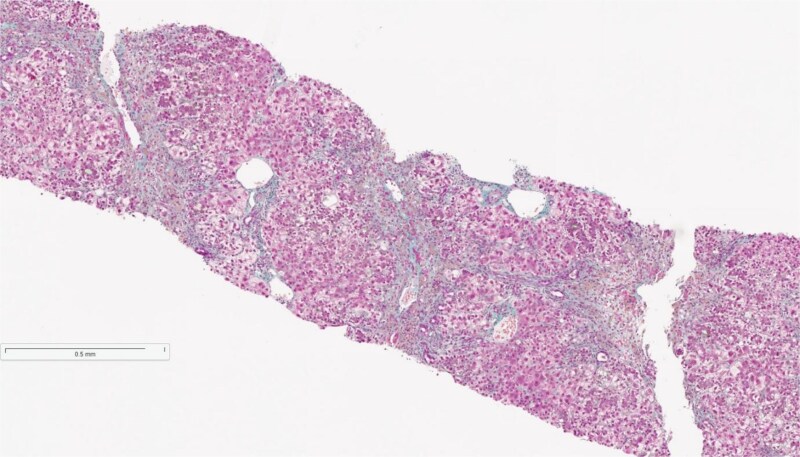
Acute hepatitis with significant cholestasis, associated collapse, and confluent necrosis. (×50 Masson trichrome).

Following this, she underwent a liver biopsy which showed overall features of severe acute hepatitis with significant cholestasis, associated collapse and confluent necrosis (fig. 1).

Given her clinical history and negative liver screen, the most likely cause of her presentation was suspected to be DILI secondary to semaglutide. To support this, we have since performed a formal assessment using the Revised Electronic Causality Assessment Method (RECAM) [6], which yielded a total score of 9, indicating that it was highly probable that semaglutide was the causative agent in her acute liver failure. As a result, semaglutide was permanently discontinued. She subsequently made a full recovery, with complete normalisation of her liver function tests.

## Discussion

GLP-1RAs associated acute liver injury is a rare entity. In large clinical trials, elevations in serum enzymes were no more common with semaglutide therapy than with placebo or comparator agents. Treatment with semaglutide and other GLP-1 analogues is often associated with improvements in serum aminotransferase levels [7], making them potential treatments for non-alcoholic fatty liver disease. However, a 2013 case report discussed drug induced liver injury from liraglutide [8] whilst a literature review identified only three cases that implicated semaglutide as the cause of drug induced liver injury [9–11]. Despite this, GLP-1Ras are not thought to have any significant hepatotoxic potential.

Going forwards, more vigiliance against the potential hepatotoxic effects of Semaglutide needs to be had with clinicians encouraged to report on adverse events relating to its use. Further research is also required in order to ascertain any potential mechanism for its effect on the liver. As the popularity and access to these medications increases via online pharmacies, patient education is needed to raise awareness of potential serious complications. This is particularly important as online prescriptions are not regulated. The patient also informed us that she never saw a clinician to obtain this medication and was started on it when she did not meet the criteria for its use. This case highlights, therefore, the potential for these medications to be inadvertently prescribed outside the licenced indication, or without adequate monitoring, leaving semaglutide and other GLP-1RAs open to abuse. This is potentially dangerous and we hypothesise that vulnerable patients, such as patients with eating disorders, could potentially utilise this to obtain these medications inappropriately. The Medicines and Healthcare products Regulatory Agency (MHRA) have recently released some cautionary advice regarding semaglutide [12]. They report that falsified preparations, some that contain insulin, have been discovered and recommend patients always obtain these medications from qualified healthcare professionals.

This case highlights the need for further research into semaglutide and drug induced liver injury. This report also highlights the need for better clinician awareness of drug induced liver injury as a potential rare side effect to ensure appropriate monitoring is happening for patients receiving GLP-1RAs.
